# Evaluation of the relation between cardiac biomarkers and thorax computerized tomography findings in COVID-19 patients

**DOI:** 10.2217/bmm-2020-0388

**Published:** 2021-01-27

**Authors:** Cigdem Ileri, Zekeriya Dogan, Beste Ozben, Cagla Karaoglu, Nuran Gunay, Kursat Tigen, Sema Basat, Cihangir Uyan

**Affiliations:** ^1^Department of Cardiology, Umraniye Research & Training Hospital, University of Health Sciences, Istanbul, Turkey; ^2^Department of Cardiology, Marmara University School of Medicine, Istanbul, Turkey; ^3^Department of Internal Medicine, Umraniye Research & Training Hospital, University of Health Sciences, Istanbul, Turkey

**Keywords:** cardiac biomarkers, COVID-19, myocardial injury, thorax CT, troponin

## Abstract

**Background:** Troponin levels may be elevated in COVID-19 infection. The aim of this study was to the explore relation between troponin levels and COVID-19 severity. **Materials & methods/Results:** One hundred and forty consecutive patients with COVID-19 pneumonia were included. Diagnosis of COVID-19 pneumonia was based on positive chest computed tomography (CT) findings. Quantitative PCR test was performed in all patients. Only 74 patients were quantitative PCR-positive. Twenty four patients had severe CT findings and 27 patients had progressive disease. These patients had significantly lower albumin and higher ferritin, D-dimer, lactate dehydrogenase, C-reactive protein, and high-sensitivity cardiac troponin I (hs-cTnI). **Conclusion:** COVID-19 patients with severe CT findings and progressive disease had higher hs-cTnI levels suggesting the use of hs-cTnI in risk stratification.

The first case of COVID-19 caused by a new coronavirus, SARS-CoV-2, was reported in Wuhan (Hubei, China) on 8 December 2019 [1]. The epidemic then spread rapidly across the world, and the WHO declared a pandemic on 11 March 2020.

A common clinical manifestation of the disease is respiratory failure due to pneumonia. Approximately 25–50% of SARS-CoV-2 infected patients have comorbidities [2]. Diabetes and cardiovascular diseases (CVDs), especially hypertension, are the most common comorbidities observed in patients with COVID-19 according to a meta-analysis [3]. The presence of concomitant cardiac disease is associated with poor prognosis, increased rate of mortality, septic shock and thromboembolic events [4].

Mild troponin elevations are frequently detected in COVID-19 patients [5]. Imbalance between oxygen demand and supply may cause myocardial injury reflected as mild troponin elevation. In an earlier study, it was reported that 12% of the COVID-19 patients had troponin elevation diagnosed as acute myocardial injury [6]. Troponin levels may be associated with disease severity [5,7]. COVID-19 may cause myocarditis and/or stress cardiomyopathy; which may explain moderate increases in troponin in these patients [8,9]. Progressive troponin increases have been suggested to predict a cytokine storm along with other biomarkers [5,10,11]. However, these classifications and predictions are mostly based on our knowledge and experience in previous viral infections.

Quantitative PCR (qPCR) is the reference standard for the diagnosis of COVID-19, but it has high false-negativity rates due to reasons such as inappropriate samples, diagnostic kit performance, viral load and sampling time [12]. On the other hand, thorax computed tomography (CT) may be a practical and reliable diagnostic method for COVID-19 with a higher sensitivity (97%) compared with PCR (59%) [13].

The aim of this study was to determine the relation between the troponin levels and COVID-19 severity defined according to thorax CT findings. We also investigated the epidemiological features, laboratory findings and comorbidities of patients who were hospitalized in a tertiary hospital in Turkey.

## Materials & methods

The investigation conformed to the principles outlined in the Declaration of Helsinki. The study was approved by the local ethics committee. All participants gave written informed consent.

One hundred and forty consecutive hospitalized COVID-19 patients between 24 March 2020 and 4 May 2020 constituted our study population. According to our national diagnostic algorithm, all patients with complaints of fever and coughing underwent thorax CT and laboratory tests including complete blood count, C-reactive protein (CRP), ferritin and D-dimer on admission. The diagnosis of COVID-19 and initiation of treatment were based on the presence of findings compatible with viral pneumonia on thorax CT and laboratory test results including lymphopenia, elevated CRP, ferritin and D-dimer. qPCR test was also performed simultaneously. While a positive qPCR test confirmed the diagnosis of COVID-19; an initial negative qPCR did not exclude COVID-19 and qPCR was repeated 24 h later. Even if the control qPCR was again negative; the patient was regarded as COVID-19 positive due to its high probability as the cause of viral pneumonia findings in thorax CT in this pandemic era and treated according to COVID-19 treatment protocols while it was advised to explore the other possible causes of viral pneumonia [14].

Patients were admitted to the hospital within 3 days after the onset of the symptoms. On admission, blood tests including complete blood count, creatinine, albumin, ferritin, D-dimer, lactate dehydrogenase (LDH), CRP, procalcitonin and high-sensitivity cardiac troponin I (hs-cTnI) were performed and were repeated to evaluate response to treatment and course of the disease.

Thorax CT findings of the patients were assessed by a clinician blinded to the clinical characteristics of the patients. The degree of involvement of each of the five lobes was classified as no involvement (0%), minimal (1–25%), mild (26–50%), moderate (51–75%) or severe (76–100%). No involvement corresponded to a lobe score of 0, minimal involvement to a lobe score of 1, mild involvement to a lobe score of 2, moderate involvement to a lobe score of 3 and severe involvement to a lobe score of 4. The total lung severity score was the sum of the five lobe scores ranging from 0 (no abnormality) to 20 (more than 75% of each lung lobe involved by COVID-19 lesion) [15,16].

An increase in densities and volumes of consolidations and ground-glass opacities, development of new consolidations at the follow-up were consistent with the radiological progression of COVID-19 [16,17]. An increase in the need for oxygen treatment and clinical deterioration of patients were accepted as clinical progression of the disease [18]. Radiological progression, clinical deterioration or rehospitalization for COVID-19 was accepted as the progression of COVID-19 in our study. Early warning score for 2019-nCoV was used to decide for admission of the patients into intensive care unit (ICU) [19].

## Statistical analysis

Statistical analyses were performed by statistical software (SPSS 11.0 for windows, IL, USA). Continuous variables were checked for normal distribution by the Kolmogorov–Smirnov test. Continuous data were expressed as mean ± standard deviation or median with 25 and 75 percentiles and interquartile range while categorical data were presented as number of patients. Chi-squared test was used for comparison of categorical variables. Student’s *t*-test or ANOVA was used to compare the normally distributed continuous variables while Mann–Whitney *U*-test or Kruskal–Wallis test was used to compare the nonparametric continuous variables. *Post hoc* analyses were performed using Bonferroni test when an overall statistical significance was determined. Logistic regression analysis was performed to explore the independent predictors of severe CT findings, progressive disease and need for ICU. Statistical significance was accepted as a p < 0.05.

## Results

One hundred and forty consecutive patients who were hospitalized in our clinic due to COVID-19 infection/pneumonia were included in the study. The mean age of the patients was 55 ± 16 years and 82 patients were male. While all the patients had positive chest CT findings compatible with COVID-19 pneumonia, PCR was positive in only 74 patients (52.9%). Elevated LDH levels (99%), elevated CRP levels (91%) and elevated D-dimer (85%) were detected in most of the patients. Lymphopenia was observed in 26% of the patients and troponin levels were elevated in 18% of the patients. There were bilateral lung infiltrations in 123 patients (87.9%) while only 17 patients (12.1%) had unilateral lung involvement. The chest CT findings were mild in 74 patients (52.9%), moderate in 42 patients (30%) and severe in 24 patients (17.1%). The general characteristics and laboratory parameters of the patients according to the severity of chest CT findings are listed in Table 1. There were not any significant differences in the frequencies of hypertension, diabetes, coronary artery disease, heart failure and renal failure among patients according to the severity of chest CT findings. Patients with severe CT findings had significantly lower albumin and higher total lung severity scores, ferritin, peak values of D-dimer and basal and peak values of LDH, CRP and hs-cTnI levels.

**Table 1.** The general characteristics and laboratory parameters of the patients according to severity of chest computed tomography findings.

Parameters	All patients (n = 140)	Mild CT findings (n = 74)	Moderate CT findings (n = 42)	Severe CT findings (n = 24)	p-value
Mean age (years) (range)	55 ± 16 (23–89)	53.5 ± 16.7 (23–89)	55.6 ± 16.5 (23–88)	58.1 ± 15.5 (30–88)	0.474
Male sex (n; %)	82 (58.6)	40 (54.1)	25 (59.5)	17 (70.8)	0.346
Hypertension (n; %)	49 (35.0)	27 (36.5)	15 (35.7)	7 (29.2)	0.802
Diabetes (n; %)	36 (25.7)	20 (27.0)	9 (21.4)	7 (29.2)	0.733
Coronary artery disease (n; %)	10 (7.1)	5 (6.8)	4 (9.5)	1 (4.2)	0.706
Heart failure (n; %)	12 (8.6)	5 (6.8)	4 (9.5)	3 (12.5)	0.660
Renal failure (n; %)	10 (7.1)	5 (6.8)	2 (4.8)	3 (12.5)	0.493
Stroke (n; %)	2 (1.4)	1 (1.4)	1 (2.4)	0	0.733
Asthma (n; %)	16 (11.4)	11 (14.9)	3 (7.1)	2 (8.3)	0.396
COPD (n; %)	8 (5.7)	2 (2.7)	5 (11.9)	1 (4.2)	0.114
PCR + (n; %)	74 (52.9)	31 (41.9)	26 (61.9)	17 (70.8)	**0.018**
Lung involvement – Unilateral (n; %) – Bilateral (n; %)	17 (12.1) 123 (87.9)	17 (23) 57 (77)	0 42 (100)	0 24 (100)	**<0.001**
Total lung severity score (range)	8.0 ± 5.7 (1–20)	4.0 ± 2.5 (1–10)	10.5 ± 4.0^†^ (6–16)	15.7 ± 4.6^†^^‡^ (9–20)	**<0.001**
Length of hospital stay (days) (range) (IQR)	9 ± 5 (3–34) (6)	6 ± 3 (3–17) (3)	10 ± 5^†^ (4–24) (7)	13 ± 7^†^^‡^ (5–34) (8)	**<0.001**
Treatment regime (n; %) – Hydroxychloroquine – Oseltamivir – Favipiravir – Azithromycin – Clarithromycin – Tocilizumab – Another antibiotic (piperacillin/tazobactam, meropenem) – Enoxaparin	139 (99.3) 81 (57.9) 29 (20.7) 108 (77.1) 6 (4.3) 4 (2.9) 10 (7.1) 104 (74.3)	73 (98.6) 36 (48.6) 4 (5.4) 51 (68.9) 1 (1.4) 0 4 (5.4) 48 (64.9)	42 (100) 26 (61.9) 11 (26.2) 37 (88.1) 3 (7.1) 1 (2.4) 2 (4.8) 36 (85.7)	24 (100) 19 (79.2) 14 (58.3) 20 (83.3) 2 (8.3) 3 (12.5) 4 (16.7) 20 (83.3)	0.638 **0.026** **<0.001** **0.045** 0.188 **0.007** 0.137 **0.025**
WBC (/μl) (25−75th percentile) (IQR)	7175 ± 3771 (4825–8325) (3500)	7461 ± 3690 (5060–8590) (3530)	6636 ± 3836 (4770–7240) (2470)	7237 ± 3961 (4380–10260) (5880)	0.527
Lymphocyte (/μl) (25−75th percentile) (IQR)	1616 ± 883 (972–2080) (1108)	1759 ± 932 (1000–2243) (1243)	1464 ± 619 (980–1955) (975)	1443 ± 1066 (682–1655) (973)	0.128
Hemoglobin (g/dl) (25−75th percentile) (IQR)	13.1 ± 1.8 (11.8–14.7) (3)	13.0 ± 1.9 (11.6–14.4) (3)	13.0 ± 1.7 (12.1–14.2) (2)	13.3 ± 1.6 (12.4–14.5) (2)	0.849
Platelet (10^3^/μl) (25−75th percentile) (IQR)	220 ± 77 (164–252) (88)	223 ± 69 (167–259) (92)	209 ± 80 (162–238) (76)	230 ± 98 (179–268) (89)	0.521
Creatinine (mg/dl) (25−75th percentile) (IQR)	1.00 ± 0.66 (0.76–1.09) (0.33)	0.94 ± 0.32 (0.75–1.04) (0.29)	1.11 ± 1.09 (0.76–1.10) (0.34)	1.02 ± 0.38 (0.74–1.23) (0.49)	0.390
Albumin (g/dl) (25−75th percentile) (IQR)	3.9 ± 0.5 (3.6–4.2) (0.6)	4.0 ± 0.5 (3.8–4.4) (0.6)	3.9 ± 0.5 (3.5–4.2) (0.7)	3.6 ± 0.6^†^ (3.3–4.0) (0.7)	**0.001**
Ferritin (μg/l) (25−75th percentile) (IQR)	433 ± 810 (72–429) (357)	319 ± 817 (39–258) (219)	398 ± 674 (82–449) (367)	779 ± 929^†^^‡^ (166–1113) (947)	**0.001**
D-dimer basal (μg/l) (25−75th percentile) (IQR)	1168 ± 1657 (435–1164) (729)	1095 ± 1756 (343–1000) (657)	1047 ± 1545 (504–909) (405)	1587 ± 1525 (727–1832) (1105)	0.392
D-dimer peak (μg/l) (25−75th percentile) (IQR)	1718 ± 2104 (536–1891) (1355)	1191 ± 1744 (383–1081) (698)	1804 ± 2110 (627–1898) (1271)	3157 ± 2451^†^^‡^ (1189–3860) (2671)	**<0.001**
LDH basal (U/l) (25−75th percentile) (IQR)	313 ± 113 (242–386) (144)	277 ± 94 (208–321) (113)	321 ± 101 (239–373) (134)	411 ± 131^†^^‡^ (338–480) (142)	**<0.001**
LDH peak (U/l) (25−75th percentile) (IQR)	400 ± 203 (296–478) (182)	340 ± 196 (264–384) (120)	421 ± 166 (331–501) (170)	547 ± 210^†^^‡^ (442–705) (263)	**<0.001**
CRP basal (mg/l) (25−75th percentile) (IQR)	3.95 ± 5.09 (0.70–5.95) (5.25)	3.02 ± 5.35 (0.20–4.05) (3.85)	3.41 ± 3.44 (1.25–4.65) (3.40)	7.74 ± 5.16^†^^‡^ (2.87–12.40) (9.53)	**<0.001**
CRP peak (mg/l) (25−75th percentile) (IQR)	7.45 ± 7.50 (1.67–11.47) (9.80)	4.67 ± 6.46 (0.65–6.28) (5.63)	8.75 ± 6.06^†^ (4.10–12.50) (8.40)	13.82 ± 8.39^†^^‡^ (8.97–17.94) (8.97)	**<0.001**
Procalcitonin basal (ng/ml) (range)	0.27 ± 1.40 (0.05–13.60)	0.27 ± 1.58 (0.05–13.60)	0.40 ± 1.47 (0.05–8.28)	0.07 ± 0.07 (0.05–0.37)	0.662
Procalcitonin peak (ng/ml) (range)	0.54 ± 2.02 (0.05–13.60)	0.47 ± 2.18 (0.05–13.60)	0.68 ± 2.09 (0.05–10.80)	0.47 ± 1.28 (0.05–5.33)	0.853
Hs-troponin I basal (ng/ml) (25−75th percentile) (IQR)	0.012 ± 0.026 (0.001–0.009) (0.008)	0.010 ± 0.022 (0.001–0.008) (0.007)	0.007 ± 0.011 (0.001–0.007) (0.006)	0.027 ± 0.046^†^^‡^ (0.003–0.048) (0.045)	**0.013**
Hs-troponin I peak (ng/ml) (25−75th percentile) (IQR)	0.015 ± 0.032 (0.001–0.012) (0.011)	0.012 ± 0.026 (0.001–0.008) (0.007)	0.009 ± 0.016 (0.001–0.010) (0.009)	0.037 ± 0.056^†^^‡^ (0.003–0.050) (0.047)	**0.008**

*Post hoc* analysis.

† Denotes statistical significance in comparison to patients with mild CT findings.

‡ Denotes statistical significance in comparison to patients with moderate CT finding.

COPD: Chronic obstructive pulmonary disease; CRP: C-reactive protein; CT: Computed tomography; Hs: High sensitivity; IQR: Interquartile range; LDH: Lactate dehydrogenase; WBC: White blood cell.

Disease progression was noted in 27 patients (19.3%) and 14 (10%) patients were taken into the ICU. The general characteristics and laboratory parameters of these patients are listed in Tables 2 & 3, respectively. The patients with progressive disease had significantly higher frequencies of diabetes and PCR positivity, higher total lung severity scores, ferritin, basal and peak values of hs-cTnI, LDH and CRP, peak procalcitonin and D-dimer levels, and lower albumin levels and lymphocyte counts compared with those without disease progression. The patients who needed ICU were elderly and had higher frequencies of diabetes, higher basal and peak values of hs-cTnI, LDH, CRP, procalcitonin and D-dimer levels, and also higher ferritin and lower albumin values.

**Table 2.** The characteristics and laboratory parameters of the patients according to disease progress.

Parameters	Patients with progressive disease (n = 27)	Patients without progressive disease (n = 113)	p-value
Mean age (years) (range)	60 ± 16 (30–87)	54 ± 16 (23–89)	0.126
Male sex (n; %)	17 (63.0)	65 (57.5)	0.606
Hypertension (n; %)	7 (25.9)	42 (37.2)	0.271
Diabetes (n; %)	11 (40.7)	25 (22.1)	**0.047**
Coronary artery disease (n; %)	3 (11.1)	7 (6.2)	0.406
Heart failure (n; %)	4 (14.8)	8 (7.1)	0.246
Renal failure (n; %)	3 (11.1)	7 (6.2)	0.406
Stroke (n; %)	1 (3.7)	1 (0.9)	0.350
Asthma (n; %)	3 (11.1)	13 (11.5)	1.00
COPD (n; %)	1 (3.7)	7 (6.2)	1.00
PCR + (n; %)	20 (74.1)	54 (47.8)	**0.014**
Lung involvement – Unilateral (n; %) – Bilateral (n; %)	2 (7.4) 25 (92.6)	15 (13.3) 98 (86.7)	0.526
Total lung severity score (range)	11.6 ± 6.6 (1–20)	7.1 ± 5.1 (1–20)	**<0.001**
Length of hospital stay (days) (range) (IQR)	14 ± 7 (4–34) (9)	7 ± 4 (3–19) (4)	**<0.001**
Treatment regime (n; %) – Hydroxychloroquine – Oseltamivir – Favipiravir – Azithromycin – Clarithromycin – Tocilizumab – Another antibiotic (piperacillin/tazobactam, meropenem) Enoxaparin	27 (100) 15 (55.6) 16 (59.3) 22 (81.5) 2 (7.4) 4 (14.8) 7 (25.9) 26 (96.3)	112 (99.1) 66 (58.4) 13 (11.5) 86 (76.1) 4 (3.5) 0 3 (2.7) 78 (69.0)	1.00 0.787 **<0.001** 0.550 0.327 **0.001** **<0.001** **0.003**
WBC (/μl) (25−75th percentile) (IQR)	7894 ± 5540 (4725–8985) (4260)	7003 ± 3219 (4900–8400) (3500)	0.907
Lymphocyte (/μl) (25−75th percentile) (IQR)	1391 ± 1060 (610–1720) (1110)	1670 ± 832 (1000–2100) (1100)	**0.024**
Hemoglobin (g/dl) (25−75th percentile) (IQR)	13.1 ± 1.5 (12.2–14.3) (2)	13.0 ± 1.9 (11.8–14.6) (3)	0.985
Platelet (10^3^/μl) (25−75th percentile) (IQR)	207 ± 73 (166–250) (84)	223 ± 78 (163–256) (93)	0.342
Creatinine (mg/dl) (25−75th percentile) (IQR)	1.07 ± 0.39 (0.74–1.44) (0.70)	0.99 ± 0.71 (0.76–1.04) (0.28)	0.147
Albumin (g/dl) (25−75th percentile) (IQR)	3.6 ± 0.7 (3.1–4.1) (1.0)	4.0 ± 0.4 (3.7–4.3) (0.6)	**0.005**
Ferritin (μg/l) (25−75th percentile) (IQR)	901 ± 1083 (134–1199) (1065)	301 ± 664 (41–333) (292)	**<0.001**
D-dimer basal (μg/l) (25−75th percentile) (IQR)	1294 ± 1417 (514–1275) (761)	1136 ± 1716 (420–1060) (640)	0.100
D-dimer peak (μg/l) (25−75th percentile) (IQR)	2555 ± 2166 (1175–3605) (2430)	1511 ± 2046 (510–1335) (825)	**0.001**
LDH basal (U/l) (25−75th percentile) (IQR)	387 ± 156 (306–489) (183)	296 ± 93 (227–343) (116)	**0.001**
LDH peak (U/l) (25−75th percentile) (IQR)	626 ± 331 (427–718) (291)	346 ± 102 (281–420) (139)	**<0.001**
CRP basal (mg/l) (25−75th percentile) (IQR)	6.25 ± 7.40 (1.30–8.60) (7.30)	3.40 ± 4.23 (0.60–5.65) (5.05)	**0.013**
CRP peak (mg/l) (25−75th percentile) (IQR)	14.26 ± 9.10 (8.90–19.30) (10.40)	5.81 ± 6.04 (1.10–8.83) (7.73)	**<0.001**
Procalcitonin basal (ng/ml) (range)	0.45 ± 1.59 (0.05–8.28)	0.24 ± 1.36 (0.05–13.60)	0.053
Procalcitonin peak (ng/ml) (range)	0.95 ± 2.32 (0.05–10.80)	0.44 ± 1.94 (0.05–13.60)	**0.001**
Hs-troponin I basal (ng/ml) (25−75th percentile) (IQR)	0.020 ± 0.043 (0.003–0.011) (0.008)	0.010 ± 0.020 (0.001–0.009) (0.008)	**0.033**
Hs-troponin I peak (ng/ml) (25−75th percentile) (IQR)	0.034 ± 0.055 (0.004–0.036) (0.032)	0.011 ± 0.022 (0.001–0.009) (0.008)	**0.002**

COPD: Chronic obstructive pulmonary disease; CRP: C-reactive protein; Hs: High sensitivity; IQR: Interquartile range; LDH: Lactate dehydrogenase; WBC: White blood cell.

**Table 3.** The general characteristics and laboratory parameters of the patients according to need of intensive care unit.

Parameters	Patients transported to ICU (n = 14)	Patients without need for ICU (n = 126)	p-value
Mean age (years) (range)	63 ± 14 (41–87)	54 ± 16 (23–89)	**0.048**
Male sex (n; %)	10 (71.4)	72 (57.1)	0.397
Hypertension (n; %)	5 (35.7)	44 (34.9)	0.953
Diabetes (n; %)	8 (57.1)	28 (22.2)	**0.005**
Coronary artery disease (n; %)	1 (7.1)	9 (7.1)	1.00
Heart failure (n; %)	2 (14.3)	10 (7.9)	0.343
Renal failure (n; %)	1 (7.1)	9 (7.1)	1.00
Stroke (n; %)	0	2 (1.6)	1.00
Asthma (n; %)	2 (14.3)	14 (11.1)	0.663
COPD (n; %)	1 (7.1)	7 (5.6)	0.579
PCR + (n; %)	10 (71.4)	64 (50.8)	0.168
Lung involvement – Unilateral (n; %) – Bilateral (n; %)	2 (14.3) 12 (85.7)	15 (11.9) 111 (88.1)	0.679
Total lung severity score (range)	13.6 ± 6.7 (1–20)	7.3 ± 5.2 (1–20)	**<0.001**
Length of hospital stay (days) (range) (IQR)	18 ± 7 (9–34) (11)	8 ± 4 (3–19) (5)	**<0.001**
Treatment regime (n; %) – Hydroxychloroquine – Oseltamivir – Favipiravir – Azithromycin – Clarithromycin – Tocilizumab – Another antibiotic (piperacillin/tazobactam, meropenem) Enoxaparin	14 (100) 8 (57.1) 10 (71.4) 12 (85.7) 1 (7.1) 4 (28.6) 7 (50.0) 14 (100)	125 (99.2) 73 (57.9) 19 (15.1) 96 (76.2) 5 (4.0) 0 3 (2.4) 90 (71.4)	1.00 0.955 **<0.001** 0.523 0.475 **<0.001** **<0.001** **0.021**
WBC (/μl) (25−75th percentile) (IQR)	7550 ± 5264 (5025–9275) (4250)	7134 ± 3593 (4875–8405) (3530)	0.900
Lymphocyte (/μl) (25−75th percentile) (IQR)	1351 ± 1073 (650–1510) (860)	1646 ± 860 (1015–2135) (1120)	0.067
Hemoglobin (g/dl) (25−75th percentile) (IQR)	13.1 ± 1.8 (11.7–14.7) (3)	13.0 ± 1.8 (11.8–14.6) (3)	0.945
Platelet (10^3^/μl) (25−75th percentile) (IQR)	192 ± 72 (147–222) (75)	223 ± 77 (171–258) (87)	0.147
Creatinine (mg/dl) (25−75th percentile) (IQR)	1.13 ± 0.40 (0.75–1.44) (0.69)	0.99 ± 0.69 (0.75–1.04) (0.29)	0.118
Albumin (g/dl) (25−75th percentile) (IQR)	3.4 ± 0.7 (2.8–3.9) (1.1)	4.0 ± 0.5 (3.7–4.3) (0.6)	**0.002**
Ferritin (μg/l) (25−75th percentile) (IQR)	1380 ± 1242 (393–2100) (1707)	300 ± 632 (48–344) (296)	**<0.001**
D-dimer basal (μg/l) (25−75th percentile) (IQR)	1583 ± 1756 (725–1842) (1117)	1120 ± 1646 (410–1103) (693)	**0.043**
D-dimer peak (μg/l) (25−75th percentile) (IQR)	3279 ± 1833 (1814–4333) (2519)	1539 ± 2065 (467–1302) (835)	**<0.001**
LDH basal (U/l) (25−75th percentile) (IQR)	373 ± 103 (308–424) (116)	307 ± 1133 (226–359) (133)	**0.009**
LDH peak (U/l) (25−75th percentile) (IQR)	769 ± 379 (499–913) (414)	359 ± 118 (274–434) (160)	**<0.001**
CRP basal (mg/l) (25−75th percentile) (IQR)	8.57 ± 9.28 (2.37–12.80) (10.43)	3.43 ± 4.15 (0.40–5.40) (5.00)	**0.010**
CRP peak (mg/l) (25−75th percentile) (IQR)	19.41 ± 9.00 (11.50–26.95) (15.45)	6.11 ± 6.01 (0.95–9.60) (8.65)	**<0.001**
Procalcitonin basal (ng/ml) (range)	0.22 ± 0.35 (0.05–1.29)	0.28 ± 1.47 (0.05–13.60)	**0.003**
Procalcitonin peak (ng/ml) (range)	1.01 ± 1.59 (0.05–5.33)	0.48 ± 2.06 (0.05–13.60)	**<0.001**
Hs-troponin I basal (ng/ml) (25−75th percentile) (IQR)	0.027 ± 0.052 (0.004–0.027) (0.023)	0.010 ± 0.021 (0.001–0.008) (0.007)	**0.004**
Hs-troponin I peak (ng/ml) (25−75th percentile) (IQR)	0.054 ± 0.066 (0.009–0.121) (0.112)	0.011 ± 0.023 (0.001–0.008) (0.007)	**<0.001**

COPD: Chronic obstructive pulmonary disease; CRP: C-reactive protein; Hs: High sensitivity; ICU: Intensive care unit; IQR: Interquartile range; LDH: Lactate dehydrogenase; WBC: White blood cell.

Two patients (1.4%) died in the ICU. They were both male and had higher basal and peak values of LDH, CRP, procalcitonin and D-dimer levels and also higher ferritin values. Their baseline hs-cTnI values were normal but peak troponin levels were higher. One of them had comorbidities like hypertension, diabetes, coronary artery disease, heart failure and renal failure with negative PCR test and bilateral moderate CT findings while the other patient had severe CT findings and positive PCR test with no comorbidities.

Logistic regression analysis did not reveal troponin levels as an independent predictor for severe CT findings, progressive disease or need for ICU when adjusted by age, sex, CRP, LDH, ferritin and D-dimer.

## Discussion

In our study, we found that patients with severe CT findings, disease progression and need for ICU had significantly higher troponin levels although the significance of hs-cTnI was lost when adjusted by age, sex and other inflammatory markers.

Troponin levels have been reported to be elevated in 8–28% of COVID-19 patients suggesting the presence of myocardial injury [20]. The release of modest levels of troponin caused by either viral or immune-mediated cardiac injury at the initial phase of the infection indicates a worse outcome [20]. Guo *et al.* have reported that myocardial injury characterized by moderate troponin elevation was mostly seen in patients with underlying CVD or other comorbidities and high levels of troponin together with natriuretic peptides on admission or at follow-up conferred up to five-times the risk of arrhythmias, mortality and ventilation need [11].

This situation may be similar to troponin elevation observed in acute respiratory distress syndrome or sepsis. Circulating troponin is detectable in over 90% of patients with acute respiratory distress syndrome and is associated with degree of critical illness [21]. While major signs of infection including tachycardia and fever increase the oxygen demand, hypotension due to cytokine storm or sepsis and pneumonia induced hypoxemia can result in insufficient supply impairing the balance of myocardial oxygen need and supply [22]. This imbalance may lead to ischemic signs and/or symptoms with a typical rise and fall pattern of troponin without classical plaque rupture and coronary thrombosis and is named as type-II myocardial infarction [23]. Patients with type-II myocardial infarction or injury usually have multiple comorbidities and poor prognosis. One of the most common precipitating factors for type-II myocardial infarction or injury is sepsis, which is originated predominantly from the lower respiratory tract. Anemia, electrolyte imbalance, arrhythmia and hypotension are also among the common factors associated with type-II myocardial infarction or injury [24]. In our study, troponin was elevated in 25 patients. The typical rise and fall pattern of troponin was observed in five patients while most of the patients (18 patients) had steady-state low positive troponin values. Two patients had continuously increasing troponin levels.

Whether the exact mechanism of myocardial injury is due to primary infection or is secondary to respiratory system involvement is still not known. It is also not clear whether it reflects a systemic or local ischemic/inflammatory process [25]. In addition, troponin elevation may result from epicardial coronary artery lesions, myocarditis or pulmonary embolism. However, continuous increase in biomarkers can be a sign of uncontrolled and amplified inflammatory response [20].

According to the data of The Chinese Center for Disease Control and Prevention for COVID-19 in mainland China, the overall case fatality rate was 2.3% (1023 deaths among 44,672 confirmed cases), and the mortality reached 10.5% in patients with an underlying CVD [1]. Mortality rate of our study was 1.4%, which was lower compared with the global data. Higher mortality rates are expected in patients with comorbidities [26]. However, in our study, one of the patients who died had CVD while the other patient had no comorbidity.

Viral load, host immune system, age and comorbidities are the major factors that lead to clinical differences [20]. One of the important results of our study was the higher frequency of diabetes among patients with progressive disease and ICU need. This result was consistent with the previous study of first 138 hospitalized patients in Wuhan [27]. Although this study pointed out cerebrovascular diseases and CVDs, especially hypertension, as important comorbidities in clinical deterioration, we did not confirm this in our study.

The most prominent laboratory changes in our study were elevations in CRP (91%), D-dimer (85%) and LDH levels (99%). According to the data obtained from the first 138 patients at the beginning of the pandemic, the most common biochemical abnormalities were lymphopenia, prolonged prothrombin time and elevated LDH [27]. Zhu *et al.* has reported biochemical findings of COVID-19 patients as elevated CRP (73.6%), elevated D-dimer levels (37.2%), lymphopenia (56.5%), elevated procalcitonin (17.5%) and leukocytosis (12.6%) [28]. Unlike the previous studies, lymphopenia was a less common finding of our study (26%). While procalcitonin levels are generally normal because of the absence of a bacterial infection, high CRP level is a common finding [28]. Liu *et al.* reported that CRP levels but not procalcitonin levels had a significant correlation with disease severity [29]. The course of the laboratory parameters during hospitalization is also related with outcome [10]. Lymphopenia is highly prognostic and recovery of lymphocyte count is related with clinical improvement [20]. D-dimer is one of the most common laboratory abnormalities of the disease and increasing levels of D-dimer are related with in-hospital mortality [30]. In our study, patients with disease progression and need of ICU had a continuous increase in hs-cTnI, CRP, procalcitonin and D-dimer levels.

## Study limitations

The major limitations of our study were the relatively small sample size and its being a single-center study. A larger cohort study is needed to verify our results. In our study, the diagnosis of COVID-19 was based on chest CT findings. These CT findings are not specific to COVID-19 disease and may be seen in other viral pneumonia cases. Thus there might be the possibility of overdiagnosis of COVID-19 in our cohort. Cardiovascular involvements of patients with elevated troponin levels could not be verified by transthoracic echocardiography or cardiac MRI due to the isolation condition in our hospital. N-terminal pro-brain natriuretic peptide levels of the patients were not assessed in our study.

## Conclusion

COVID-19 patients with severe CT manifestations, disease progression and need for ICU have significantly higher troponin levels in addition to elevations in ferritin, D-dimer LDH and CRP suggesting the use of troponin levels in the risk stratification of COVID-19 patients. There is need for further studies with larger sample sizes to understand the associations between high troponin and cardiovascular complications of COVID-19.

**Summary points**

- Troponin levels may be elevated in patients with COVID-19.
- Patients with severe computed tomography findings had significantly higher troponin levels in addition to higher total lung severity scores, ferritin, D-dimer, lactate dehydrogenase, and C-reactive protein and lower albumin levels.
- Patients with disease progression and need for intensive care unit had also significantly higher troponin levels in addition to higher total lung severity scores, ferritin, D-dimer, lactate dehydrogenase, and C-reactive protein and lower albumin levels.
- The significance of troponin on predicting disease severity or progression is lost when adjusted by age, sex and other biochemical markers.
